# Transcriptome Dedifferentiation Observed in Animal Primary Cultures is Essential to Plant Reprogramming

**DOI:** 10.26502/jbsb.5107039

**Published:** 2022-10-07

**Authors:** Norichika Ogata

**Affiliations:** Nihon BioData Corporation, Kawasaki, Japan

**Keywords:** Dedifferentiation, Information Entropy, Liberality, Primary Culture, Transcriptome, Reprogramming

## Abstract

Tissue culture environment liberate cells from ordinary laws of multi-cellular organisms. This liberation enables cells several behaviors, such as growth, dedifferentiation, acquisition of pluripotency, immortalization and reprogramming. Each phenomenon is relating to each other and hardly to determine. Recently, dedifferentiation of animal cell was quantified as increasing liberality which is information entropy of transcriptome. The increasing liberality induced by tissue culture may reappear in plant cells too. Here we corroborated it. Measuring liberality during reprogramming of plant cells suggested that reprogramming is a combined phenomenon of dedifferentiation and re-differentiation.

## 1. Introduction

Tissue culture is performed to maintain isolated portions of multicellular organisms in an artificial milieu that is outside the individual organism and for considerable periods of time [1]. It is known over a century that cells derived from cultured explants are, in general, different from cells of the corresponding tissue in a living organism [2,3]. In these tissue cultures, cells are liberated from stimulations and prohibition which is ordinary in multi-cellular organisms [4]. This liberation is essential for growth, dedifferentiation, acquisition of pluripotency, immortalization and reprogramming. However, each phenomena related to each other and some of them had not been scientifically justified, such as dedifferentiation, reprogramming and immortalization. For example, it is not unclear that whether the immortalized cell line has individual cellular immortality or population immortality with gene pool sharing. In other case, historically, proliferations of cultured cells were considered to a result of dedifferentiation [2]. To concrete discussion, concrete definition of each phenomenon which happens in liberated tissue cultures. Recently, the cellular dedifferentiation was quantitive defined as increasing of information entropy of transcriptome [5]. A dedifferentiation of animal cells in primary explant culture was corroborated previously [5]. Then we hypothesized that dedifferentiation of cells in primary explant culture is a common phenomenon for diverse multi-cellular organisms. Here we corroborated whether plant cell dedifferentiated in primary explant culture too or not using a shared transcriptome data set [6].

## 2. Materials and Methods

Transcriptome data set was obtained from DDBJ SRA (DRA000400) [6]. In this entry, time course total RNA sampling during reprogramming of leaf cells of the Moss Physcomitrella patens (0, 1, 3, 6, 12 and 24 hours). Each sample has there biological replicates. We mapped transcriptome sequence data using bowtie 1.1.2 [7] since they used SOLiD sequencer. Information entropy was calculated from all count data as previously described [5]. We compared culture time and information entropy of transcriptome data.

## 3. Results and Discussion

**Figure 1:**
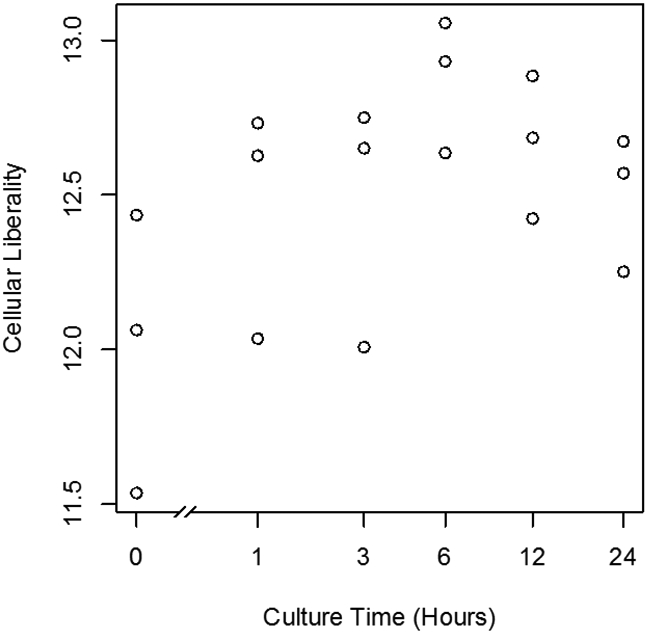
Scatter plot of culture time vs cellular liberality. The moss leaf cells were cultured in BCDAT medium. Liberalities, the information entropy of transcriptome were measured in each culture time.

**Figure 2:**
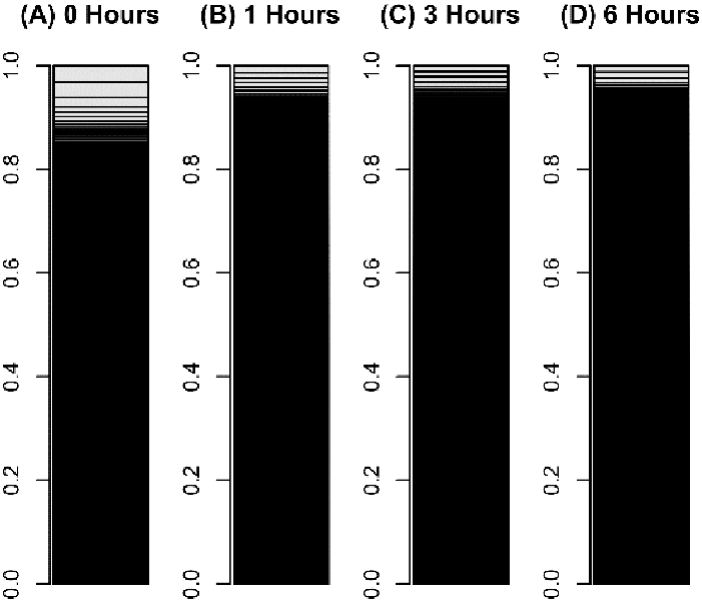
Bar charts of leaf cells transcriptome during reprogramming. The occupation rate of genes in a transcriptome was plotted in a bar chart. Heights of boxes in a bar chart indicate the occupation rate of genes in a transcriptome. Although more than 50,000 transcripts are included in these bar charts, most are invisible and are included in black regions. (A) A transcriptome of leaf cells cultured for 0 hours in BCDAT medium. (B) A transcriptomes of leaf cells cultured for 1 hour in BCDAT medium. (C) A transcriptomes of leaf cells cultured for 3 hours in BCDAT medium. (D) A transcriptomes of leaf cells cultured for 6 hours in BCDAT medium.

The plant cells dedifferentiated in primary explant culture, equal to animal cells; the information entropy of transcriptome data increased during culture (Figure 1, Figure 2). This result suggested that dedifferentiation of cells in primary explant culture is a common phenomenon for diverse multi-cellular organisms. However, in the case of plants, the information entropy decreases after the information entropy increases up to about 6 hours. This is thought to be re-differentiation to construct new plants and reprogramming can be explained as an integration of dedifferentiation and regeneration. Callus may be regarded as dedifferentiated cells which holding down the re-differentiation process; using callus and plant tissue culture, bi-/multi-stability of transcriptome [8, 9] could be demonstrated in plant. If their re-differentiation process is caused by the determination of intercellular division of labor based on cell-to-cell communication [10], it will be possible to examine them in a test that separates cultured cells. Cellular dedifferentiation and differentiation have been understood as the direction of cellular morphology and phenotype change [2,11]. In this decade, several studies [12-15] following our research [5] repeatedly measured the degree of cellular dedifferentiation and differentiation as a transcriptome Shannon entropy. The Shannon entropy is a kind of alpha diversity in ecology [16], and the transcriptome Shannon entropy is simply transcriptome diversity [5, 17]. It is not incorrect to call it the alpha diversity of the transcriptome, but that would leave its biological significance undefined, as would each principal component that came up in the principal component analysis. Since we can quantitatively assess, judge, and define that dedifferentiation is an increase in the Shannon entropy of the transcriptome and differentiation is a decrease in the Shannon entropy of the transcriptome, it is more accurate to position the “value of information entropy of the transcriptome” not as a mere bioinformatics measure; however, as a number with obvious biological and bioengineering significance, such as viable cell rate, cell density, specific growth rate, or pcd (pg/cell/day). Here we call the quantitative value of cellular dedifferentiation and differentiation “liberality,” since a previous study explained the changes were happening to cultured cells as “libère” [18].

## References

[1] Murray Margaret Ransone GK. A Bibliography of the Research in Tissue Culture, 1884-1950. New York: Academic Press (1953): 1741.

[2] Champy C. Quelques résultats de la méthode de culture de tissus. I. Généralités. II. Le muscle lisse. . Archives de zoologie expérimentale et générale 53 (1913-1914): 42–51.

[3] Carleton HM. Tissue culture: A critical summary. Journal of Experimental Biology 1 (1923): 131–151.

[4] Canguilhem G. La connaissance de la vie. Paris: Librairie Philosophique J Vrin (1965).

[5] Ogata N, Yokoyama T, Iwabuchi K. Transcriptome responses of insect fat body cells to tissue culture environment. PLoS One 7 (2012): e34940. 22493724 10.1371/journal.pone.0034940 PMC3321044

[6] Nishiyama T, Miyawaki K, Ohshima M, Digital gene expression profiling by 5'-end sequencing of cDNAs during reprogramming in the moss Physcomitrella patens. PLoS One 7 (2012): e36471. 22574165 10.1371/journal.pone.0036471 PMC3344888

[7] Langmead B, Trapnell C, Pop M, Ultrafast and memory-efficient alignment of short DNA sequences to the human genome. Genome Biol 10 (2009): R25. 19261174 10.1186/gb-2009-10-3-r25 PMC2690996

[8] Ferrell JE Jr. Bistability, bifurcations, and Waddington's epigenetic landscape. Curr Biol 22 (2012): R458–466. 22677291 10.1016/j.cub.2012.03.045 PMC3372930

[9] Ogata N, Kozaki T, Yokoyama T, Comparison between the Amount of Environmental Change and the Amount of Transcriptome Change. PLoS One 10 (2015): e0144822. 26657512 10.1371/journal.pone.0144822 PMC4678807

[10] de la Cova C, Abril M, Bellosta P, Drosophila myc regulates organ size by inducing cell competition. Cell 117 (2004):107–116. 15066286 10.1016/s0092-8674(04)00214-4

[11] Carleton HM, Tissue culture: A critical summary. J. Exp. Biol 1 (1923): 131–151.

[12] Guo M, Bao EL, Wagner M, SLICE: determining cell differentiation and lineage based on single cell entropy. Nucleic Acids Res 45 (2017): e54. 27998929 10.1093/nar/gkw1278 PMC5397210

[13] Teschendorff AE, Enver T. Single-cell entropy for accurate estimation of differentiation potency from a cell’s transcriptome, Nat Commun 8 (2017): 15599. 28569836 10.1038/ncomms15599 PMC5461595

[14] Wiesner K, Teles J, Hartnor M, Haematopoietic stem cells: entropic landscapes of differentiation. Interface Focus 6 (2018): 20180040. 10.1098/rsfs.2018.0040 PMC6227807 30443337

[15] Kannan S, Farid M, Lin BL, Transcriptomic entropy benchmarks stem cell-derived cardiomyocyte maturation against endogenous tissue at single cell level. PloS Comput Biol 17 (2021): e1009305. 34534204 10.1371/journal.pcbi.1009305 PMC8448341

[16] Dongmei A, Ruocheng H, Jin W, Integrated metagenomic data analysis demonstrates that a loss of diversity in oral microbiota is associated with periodontitis. BMC Genomics 18 (2017): 1041. 28198672 10.1186/s12864-016-3254-5 PMC5310281

[17] Martinez O, Reyes-Valdes MH. Defining diversity specialization and gene specificity in transcriptomes through information theory, Proc. Natl. Acad. Sci. U.S.A 105 (2008): 9709–9714. 18606989 10.1073/pnas.0803479105 PMC2443819

[18] Canguilhem G. La connaissance de la vie, Pari: Hachette (1952): 41.

